# Promising treatments of tomorrow for multiple sclerosis

**DOI:** 10.4103/0972-2327.58279

**Published:** 2009

**Authors:** Daniel M. Harrison, Peter A. Calabresi

**Affiliations:** Johns Hopkins Multiple Sclerosis Center, Johns Hopkins University School of Medicine, Baltimore, Maryland, USA

**Keywords:** Drugs, investigational, multiple sclerosis, therapeutics

## Abstract

The therapeutic options for multiple sclerosis are rapidly expanding. What was once seen as a disease with little hope for treatment is now a target of rapid drug development. Current therapies have demonstrated efficacy in limiting the impact of the disease, but none is fully effective in all patients. However, promising new treatments are on the horizon. In this review we will discuss potential novel immunomodulating drugs that are in advanced stages of investigation; these drugs include monoclonal antibodies, chimeric molecules, and oral therapies. The use of hematopoietic stem cells will also be discussed and, in addition, we will look farther ahead at possible novel targets for the development of new immunomodulatory or neuroprotective pharmaceuticals.

## Introduction

The treatment of multiple sclerosis (MS) has changed rapidly in the last two decades. With the introduction of interferon-β, and later glatiramer acetate, began an era of hope for what had previously been a mostly untreatable disease. These disease-modifying therapies have demonstrated efficacy in reducing relapse rates and MRI lesion burden, as well as in delaying the accumulation of disability.[1–5] Nevertheless, the degree to which these medications can alter the natural history of this disease is relatively modest. Newer therapies, such as the monoclonal antibody natalizumab, appear to have a more robust effect on relapse rates and MRI activity.[6] However, natalizumab has been associated with rare, but serious, complications such as the development of progressive multifocal leukoencephalopathy (PML).[7]

It is clear that there is room for newer, more effective therapies in MS, perhaps with less serious side effects. This need has stimulated a great deal of pharmaceutical research. Years of diligent investigations by the pharmaceutical industry and at academic centers has led to the development of a plethora of new agents, many of which appear promising and are in advanced stages of clinical trials. It appears likely that the next decade will see the number of treatment options in MS increase rapidly, hopefully to the benefit of patients with this disease.

In this review, we aim to discuss medications and approaches that show promise for the future. Due to the large number of such therapies, a discussion of all options is beyond the scope of this manuscript. Therefore, we will concentrate on medications that are in advanced stages of clinical trials and conclude with a summary of future prospects.

## Monoclonal Antibodies

As a means of targeting specific components of molecular pathways, monoclonal antibodies have recently become a part of the treatment in a large number of diseases, especially in the fields of oncology and autoimmune disorders. Natalizumab was the first such medication approved for use in MS, but there are now multiple promising monoclonal antibodies on the horizon.

One such medication is rituximab. This chimeric monoclonal antibody targets the CD20 molecule, leading to depletion of B-lymphocytes through antibody-dependent and complement-mediated cytotoxicity as well as by promotion of apoptotic mechanisms.[8] Rituximab has been used in non-Hodgkin type B-cell lymphoma for over a decade,[9] but is now being studied in a number of autoimmune conditions. In a 72-week, phase I, open-label study, rituximab was well tolerated by patients with relapsing–remitting MS, with few side effects and no serious infections.[10] Although there was no placebo group for comparison, this study also showed a reduction in relapse rates and decrease in the number of new gadolinium-enhanced lesions on MRI during the study period. A later placebo-controlled phase II trial demonstrated similar MRI findings, with a decreased number of cumulative new gadolinium-enhanced lesions throughout a 24-week evaluation period as compared to the placebo group (*P* < 0.001).[11] In addition, the proportion of relapse-free patients was higher in the treatment group than in the placebo group (40.0% *vs* 20.3%, *P* = 0.04). The success of these trials has led the developers of this drug to begin MS treatment trials with ocrelizumab, a fully humanized anti-CD20 monoclonal antibody thought to have less likelihood of anti-idiotypic antibody formation and infusion reactions.[12] Regardless of the results of such trials, however, the fact that PML and other infections have been described in patients receiving rituximab for lymphoma and rheumatic diseases will likely translate into guarded use of this class of immunosuppressive drugs.[13,14]

Alemtuzumab has also shown promise in the treatment of MS. This monoclonal antibody targets the CD52 molecule on lymphocytes and monocytes, leading to profound lymphocyte suppression.[15] Initial studies with this medication in secondary progressive MS were unable to demonstrate an effect on disability progression.[16] However, a more recent phase 2 trial in relapsing–remitting patients yielded positive results.[17] In this study, patients were randomized to receive treatment with either interferon β-1a or one of two doses of alemtuzumab. When compared with interferon β-1a, alemtuzumab reduced the relapse rate by 74% (hazard ratio (HR): 0.26, *P*<0.001) and reduced the risk of sustained disability by 71% (HR: 0.29, *P* < 0.001). Alemtuzumab also demonstrated superiority on MRI, with a greater reduction in T2 lesion load (*P* = 0.005) and less atrophy on T1 images (*P* = 0.02). Unfortunately, there were a series of serious side effects seen in this trial that will likely greatly limit broad use of this medication. Immune thrombocytopenic purpura developed in six patients on alemtuzumab and resulted in one death due to brain hemorrhage. Additionally, thyroid complications were seen in 22.7% of patients on alemtuzumab, of which 96% were associated with antithyroid antibodies. In fact, previous use of this drug has shown that there is a tendency for the occurrence of adverse events involving autoimmunity; examples include thyroid disease and renal failure due to anti-glomerular basement membrane disease.[18] It is postulated that the early recovery of B-cells, as compared to the later recovery of T-cells, after alemtuzumab leads to an imbalance that favors development of unregulated antibody-mediated autoreactivity.[19,20] Because of its profound effect on the disease course it is likely that there will be further investigation and use of this medication in MS, but the risks of these side effects will have to be balanced against the potential benefits to patients.

Daclizumab is a monoclonal antibody directed against the CD25 molecule, which is the alpha chain of the interleukin-2 (IL-2) receptor.[21] This antibody blocks the ability of IL-2 to bind to the IL-2α receptor. The higher expression of the IL-2β and γ receptors on natural killer T-cells seems to promote their expansion and this may have regulatory properties. This medication is approved for use in the treatment of renal allograft rejection[22] and is currently being investigated in MS. In an open-label phase II trial, patients who were deemed as ‘interferon failures’ had daclizumab added to their regimen and attempts were made to transition to monotherapy with this medication.[23] Compared to a pretreatment baseline evaluation period, the number of total and new contrast-enhanced lesions on MRI was reduced (*P* < 0.001), as were the number of relapses (*P* < 0.001) and the expanded disability status scale (EDSS) score (*P* < 0.01). The drug was relatively well tolerated and there were no serious side effects. A safety evaluation of 55 patients on this medication at the Brigham and Women's Hospital in Boston, Massachusetts, found similar tolerability to the drug amongst most patients, though two patients in this study developed cardiotoxicity.[24] Cardiotoxicity has not been previously described for this medication and, because of the open-label nature of this evaluation, it is unclear if this side effect can be directly attributed to the medication. In fact, due to the open-label nature of both of these studies, the efficacy of this medication compared to placebo or standard treatment has not yet been established. However, placebo-controlled phase II dose-finding studies with a modified anti-CD25 monoclonal antibody are near completion.

## Chimeric Molecules

A number of non-monoclonal biologic molecules have been created as means of targeted therapy for a series of autoimmune conditions. CTLA4Ig (abatacept) is once such medication. CTLA4Ig is a chimeric molecule composed of a human CD152 molecule and an IgG tail. The CD152 domain binds to B7-1 (CD80) and B7-2 (CD86) on antigen-presenting cells, blocking their ability to bind to CD28 on T-cells, which would otherwise lead to T-cell activation. This medication has already been approved for use in rheumatoid arthritis. In a phase I open-label study, 16 patients with relapsing–remitting MS were treated with one dose of CTLA4Ig in order to assess the safety of this drug.[25] Four additional subjects received four subsequent monthly doses. The drug was well tolerated, with minimal side effects. The initial attempt at a phase II trial of this medication in MS was aborted due to imbalances in the study groups after randomization and therefore the efficacy of this drug is not yet known.

Another chimeric molecule receiving attention is atacicept. This medication is a fusion between the B lymphocyte stimulator (BLyS)/APRIL (a proliferation-inducing ligand) binding portion of TACI, a TNF-related receptor, and the Fc portion of human IgG. By binding available BLyS and APRIL, this drug results in a decrease in the number of circulating mature B-cells and some plasma cells which, in turn, decreases immunoglobulin synthesis. Phase I studies have been conducted in patients with rheumatoid arthritis and systemic lupus erythematosus, which have demonstrated safety and biological plausibility.[26,27] A phase II study in MS is currently being conducted and the results are expected in 2010.[28]

## Oral Therapies

One of the major limitations of the existing treatment for MS is the route of administration. All currently approved therapies for this disease require intramuscular, subcutaneous, or intravenous administration. The lifestyle burden and discomfort that self-injections involve is a major reason for poor adherence to prescribed therapy. Additional patient education and nursing care is often required for proper administration of subcutaneous and intramuscular medications. Additionally, intravenous therapies are prone to infusion reactions and thus require monitored infusion settings. Thus, oral therapy would not only provide significant improvement in patient lifestyle and morale but would also lead to less utilization of healthcare resources and funds. Because of this obvious need, a great deal of research has been conducted in this area and there are now a number of promising oral agents in advanced stages of clinical trials.

Oral fingolimod (FTY720) is currently in phase III evaluation in relapsing–remitting and progressive forms of MS. This compound acts as a superagonist of the sphingosine-1-phosphate receptor on lymphocytes, causing aberrant internalization of the receptor.[29] Since this receptor is necessary for proper egress of these cells from secondary lymphoid tissues, a significant number of lymphocytes become trapped in the lymph nodes.[30] It appears that this effect is selective for naïve and central memory T-cells (both CCR7+), while effector memory T-cells (CCR7−) are less affected and some continue to circulate.[31] The reduction in numbers of lymphocytes leads to reduced trafficking into the central nervous system.

In a double-blind, placebo-controlled, phase II trial, fingolimod demonstrated a good safety profile and significant efficacy.[32] The primary evaluation was a placebo-controlled 6-month study, followed by another 6 months of a non-placebo-controlled extension phase. The total cumulative number of gadolinium-enhanced lesions on MRI was reduced in both tested doses of fingolimod as compared to placebo (*P* < 0.001 for the 1.25-mg dose and *P* = 0.006 for the 5.0-mg dose). The annualized relapse rate showed a relative reduction of 53% in the 5.0-mg group (*P* = 0.01) and 55% in the 1.25-mg group (*P* = 0.009). However, there were no significant differences between placebo and treatment groups in EDSS score at 12 months. Minor infections such as nasopharyngitis and influenza were more common in the treatment arms but did not lead to serious adverse events. There were two infectious adverse events that led to discontinuation of the study drug in the extension portion of the study – a case of facial herpes zoster and a case of enterocolitis. The study drug was also discontinued in one patient on the 5.0-mg dose who developed a syndrome consistent with posterior reversible leukoencephalopathy, which has also been described in other immunosuppressive agents.[33] A transient reduction in heart rate was noted with the first dose only, as was an early reduction in forced expiratory volume and forced vital capacity, both of which had been seen in phase I evaluations and neither of which led to serious adverse events. A later publication of 24-month data on a further extension of this study showed continued benefit to patients in terms of less MRI activity and lower relapse rate and, additionally, there were no further serious adverse events in the safety evaluation.[34] Preliminary data from a head-to-head study of FTY-720 *vs* Avonex^®^ was recently distributed via a manufacturer's press release.[35] This study has shown a significant reduction in relapse rate for two different doses of FTY-720 as compared to Avonex. Unfortunately, two cases of fatal central nervous system (CNS) herpes virus infections were reported in patients taking the higher dose. Two large phase III studies in relapsing–remitting MS are presently underway (the first is due to be completed in 2010) and a trial in primary progressive patients is planned.[36,37]

Cladribine is another oral medication that may soon come into use in patients with MS. Cladribine is a purine analog that is phosphorylated by deoxycytidine kinase, an enzyme mostly found in lymphocytes.[38,39] The phosphorylated drug leads to disruption of cellular metabolism and damage to DNA, ultimately leading to cell death. This has its most profound effect on CD4+ T-cells.[40] An oral formulation of this drug has recently been developed and tested, although much of the reported data are from studies of intravenous or subcutaneous formulations.[41]

In the 1990s, two placebo-controlled trials of cladribine in patients with progressive forms of MS produced slightly differing results. The first trial evaluated 51 patients receiving either four monthly intravenous doses of cladribine (0.7 mg/kg) or placebo infusions.[42] This study demonstrated less accumulation of disability (as measured by EDSS) (*P* < 0.01) and lower T2 lesion volumes (*P* < 0.02) at 12 months when compared to the placebo group. In a larger study, 159 patients received subcutaneous cladribine injections or placebo.[43] At the end of 12 months, there were no significant differences in the groups in terms of disability scores, but there was a reduction in the number and size of gadolinium-enhanced MRI lesions (*P* = 0.003). In both studies, the side effects were mild to moderate and no severe adverse events were reported.

In a placebo-controlled trial of subcutaneous cladribine in patients with aggressive relapsing–remitting MS, the relapse rates over a period of 7–18 months were 0.66/year (95% CI: 0.37 to 1.05) for cladribine and 1.34/year (95% CI: 0.90 to 1.93) for placebo.[44] Additionally, the cladribine group had significantly fewer gadolinium-enhanced lesions on MRI compared to the placebo group (*P* = 0.001). In this study, as in others, the primary adverse effect was myelosuppression, which can persist for up to 8 months post treatment.[40] A phase III trial of the oral formulation of this drug was recently completed and preliminary reports have revealed a significant reduction in the relapse rate favoring cladribine-treated patients.[45]

Another potential oral agent is laquinimod, which is structurally similar to a medication previously considered promising, i.e., roquinimex. Roquinimex demonstrated efficacy in phase II and phase III trials in MS, but investigation was halted due to the appearance of serious side effects, such as serositis and myocardial infarction, which precluded its use.[46] Laquinimod was later selected for development because of its structural similarity to roquinimex and superior efficacy in experimental autoimmune encephalitis (EAE).[47] Additionally, this medication did not show a propensity for causing tissue inflammation in other animal models.[48] In one randomized trial, 209 patients received either 0.1 mg or 0.3 mg of laquinimod or placebo for 24 weeks.[49] The primary outcome measure of this trial was satisfied for the 0.3-mg group only, with a 44% reduction in active MRI lesions in the treatment groups compared to placebo (*P* = 0.0498). Although there was a trend towards lower numbers of MRI lesions in the 0.1-mg group, this did not reach statistical significance. Additionally, compared to placebo group, there were no differences noted in relapse rates or disability measures at the end of the trial. A later phase II randomized trial utilized higher doses, with laquinimod 0.6 mg and laquinimod 0.3 mg being compared to placebo.[50] In this trial, the primary outcome was only satisfied for the 0.6-mg group, where there was a 40.4% reduction in new active MRI lesions compared to placebo (*P* = 0.0048). No statistically significant differences were seen in the MRI outcomes for the 0.3-mg group in this trial, and although suggestive trends were noted, there were no clear improvements in the clinical outcome measures in this trial either. Although no clinical improvements were seen in either trial, it should be noted they were not powered to show such differences. Thus, based on the efficacy shown by laquinimod in suppressing MRI activity in these trials, a phase III trial has been undertaken and is expected to be completed in 2011.[51]

Teriflunomide is an oral immunomodulator that exerts its effect by inhibiting pyrimidine synthesis in T-cells and other rapidly dividing cell populations.[52] It has been demonstrated to be capable of suppressing inflammatory activity in animal models of MS.[53] In a phase II trial in human subjects, 179 patients were assigned to receive 7 mg or 14 mg of teriflunomide or placebo for 36 weeks.[54] This study demonstrated a significant reduction in the number of new contrast-enhanced lesions in the treatment groups as compared to the placebo group (*P* < 0.005). Additionally, the proportion of patients with sustained EDSS increases of 1.0 or more was lower in the 14-mg group than in the placebo group (7.4 *vs* 21.3%; *P* < 0.04). Trends towards lower relapse rates were also noted but this did not reach statistical significance. The medication was generally well tolerated and a phase III evaluation is currently underway.[55] Of note, however, is the finding that teriflunomide takes an extremely long time to be cleared from the body and carries serious risk for men and women considering reproduction.

An oral formulation of the compound dimethyl fumarate, labeled BG00012, is another promising oral agent that may have both anti-inflammatory and neuroprotective effects. This drug likely activates the nuclear factor E2–related factor-2 (NrF2) pathway. Activation of the NrF2 pathway may defend against oxidative stress–induced cell death, support the blood–brain barrier, support myelin integrity, and inhibit cytokine and adhesion molecule expression. In a large trial in patients with relapsing–remitting MS, 257 patients were randomized to receive one of three doses of BG00012 or a placebo for 48 weeks.[56] The highest dose tested (240 mg) resulted in significant reductions in MRI disease activity, but the lower doses did not have this effect. The changes in clinical outcomes did not reach statistical significance. The medication was generally well tolerated, with flushing and gastrointestinal side effects being most common. A phase III study of this medication is in process and is projected to be completed in 2011.[57]

## Hematopoietic Stem Cells

All current pharmacologic therapies available for the treatment of autoimmune disease aim to modulate an aberrant immune system but none are capable of complete suppression of all abnormal activity. The integration of hematopoietic stem cell transplantation into medical regimens for autoimmune disease has been investigated as a means for complete reversal of the autoimmune response. This approach is based on a theoretical belief that if all differentiated immune cells can be removed by immunoablative chemotherapeutics, then a ‘new’ immune system can be reconstituted from hematopoietic stem cells. As in the similar treatment of some hematologic malignancies, it is hoped that this reconstituted immune system will not be prone to the same aberrant activity as its predecessor. This has been tested via a number of differing methodologies in patients with MS. In one study performed in Italy, 10 patients with secondary progressive MS received a mobilization regimen of high-dose cyclophosphamide (4 g/m^2^) and granulocyte-colony-stimulating factor (G-CSF), followed by the BEAM conditioning regimen (carmustine, etoposide, cytosine-arabinoside, and melphalan).[58] This was then followed by autologous peripheral blood stem cell transplantation. Within 4 months of treatment, all patients had complete suppression of MRI activity, with no new gadolinium lesions appearing during the follow-up period (median follow-up: 15 months; range: 4–30 months). Also, nine of the 10 patients had no new T2 lesions during the follow-up period. No major adverse events were recorded and EDSS scores remained stable during follow-up.

In a larger, multicenter study, 85 patients with progressive forms of MS were treated with a regimen similar to that described above.[59] Confirmed progression-free survival was seen in 74% (±12%) at 3 years, and post-transplant gadolinium-enhanced lesions on MRI occurred in only 8% of cases. However, this study showed relatively significant toxicity, with 15% of patients having serious infections, allergic events, or severe G-CSF-related bone pain. Additionally, there were five patients who died of procedure-related causes.

An alternate approach, in which high-dose cyclophosphamide is used and the immune system allowed to reconstitute naturally, has also been tested.[60,61] In one study by Krishnan *et al*, nine patients with aggressive relapsing–remitting MS received a short, but high-dose, pulse of cyclophosphamide at a dose of 50 mg/kg/day for 4 days, followed by treatment with G-CSF. No transplantation procedure was utilized. Since cyclophosphamide by itself should spare hematopoietic stem cells, immune reconstitution can occur from these cells without the need for transplantation. This may avoid some of the morbidity and mortality associated with transplantation that was noted in the previous study and also avoid the possibility of reintroducing pathogenic lymphocytes with the autologous marrow stem cells. There were no deaths or unexpected serious adverse events with this protocol. Mean reduction in EDSS score was 2.11 (SD: 1.97; *P* = 0.02) and there was an 81.4% reduction in gadolinium-enhanced lesions on MRI (*P* = 0.01). Two patients did require rescue treatment for clinical worsening during the study, but the remainder of the cohort remained stable.

The high-dose, pulse cyclophosphamide treatment approach may achieve a similar immunologic effect as the transplantation protocols, but it may have fewer side effects and may therefore be more likely to gain wider usage. However, further testing on a larger scale is necessary before such an approach becomes accepted as a standard therapy.

## Future directions

Based on the significant number of novel agents that are presently in the later stages of testing, the armamentarium of the physician treating patients with MS is likely to substantially increase in the next decade. Despite the possibility that we may wind up with an overwhelming number of choices, a great deal of research is continuing in laboratories around the world searching for new therapeutic targets for this and other autoimmune diseases.

The search for novel therapeutic targets has been enhanced by rapid advances in genetics and proteomics. Analysis of gene transcripts found in MS lesions via gene chip microarrays has revealed significant expression of osteopontin and aB crystallin.[62] Osteopontin has properties of a proinflammatory cytokine and may prevent the apoptosis of autoreactive T-cells.[63] On the other hand, aB crystallin has an inhibitory effect on autoimmunity and it appears to be a major target of autoreactive T-cells and intrathecal antibodies.[64] Therapeutic interventions that block osteopontin function or enhance aB crystallin function are now in early stages of development.

Proteomic techniques also show promise in the search for novel therapeutic targets. One of the more unexpected findings thus far with this technique has been the significant representation of proteins involved in the coagulation cascade within MS lesions.[65] From this analysis, tissue factor and protein C inhibitor (PCI) appear to be the most promising targets. Tissue factor may exert an effect on MS lesions via its activation of thrombin (which has proinflammatory effects), while PCI inhibits the protein C pathway. Due to these findings, hirudin, a thrombin inhibitor, and activated protein C have both been tested in EAE, with good results.

Another novel target under investigation is the voltage-gated potassium channel Kv1.3, which is specifically upregulated on effector T-cells. Kv1.3 activity appears to be critical for the function of effector memory T-cells, while central memory T-cells signal through separate calcium-dependent potassium channels: KCa3.1.[66] Since effector memory T-cells are implicated in autoimmune disorders and are abundantly present in MS brain tissue, targeting the Kv1.3 receptor may be a means of achieving more specific immunosuppression, without causing inhibition of normal immune function. Preliminary work with Kv1.3-inhibiting pharmacologic agents have supported this hypothesis thus far,[67] but much research has to be done before this approach can be tested in human subjects.

In addition to the search for new immunomodulating agents a great deal of research is being conducted in order to find therapies that may prevent the degeneration of axons and/or promote remyelination. Because of the lack of efficacy of immune-based therapies in progressive forms of MS, these approaches are most critical for patients with these subtypes. Although no axonal protective or regenerative agents are yet available, there are a few lines of research being pursued that may someday make such an approach a reality.

The myelin-associated glycoprotein (MAG) is a strong candidate for interventions that may lead to prevention of axonal degeneration. MAG is a component of myelinated internodes that is known to be involved in the regulation of axonal caliber. The observation that MAG knockout mice are prone to axonal degeneration has led to interesting observations that have implications for clinical interventions in MS.[68] It now appears that MAG is critical for axonal stability and that addition of MAG to cell culture can promote resistance to axonal degeneration. Future therapies may act by enhancing the pathways by which MAG exerts this effect on axons, thus reducing the neurodegenerative component of MS.

One potential target for promotion of remyelination is the LINGO-1 (leucine-rich repeats and Ig domain–containing neurite outgrowth inhibitor receptor–interacting protein-1) protein. It has been recently discovered that this molecule is a potent inhibitor of oligodendrocyte progenitor cells and thus an inhibitor of myelination.[69] This process may actually be inhibited within MS lesions, thus curbing post-inflammatory remyelination.[70] Experimental evidence is beginning to accumulate supporting the effects of LINGO-1 antagonists in promoting axonal remyelination, regeneration, and even functional recovery in EAE.[71] These findings are promising because, if this pathway can be properly harnessed, remyelination may one day become a key component of neurological recovery after relapses or in the setting of disability progression.

Overall, the future appears bright for patients with MS. If the current momentum continues, the next decade will likely see more specific immunomodulators being utilized, perhaps along with other drugs that will prevent axonal degeneration and stimulate repair of damaged axons. Until these agents have been fully evaluated, however, we should remain cautiously optimistic, as the adverse effects of these mediations may not truly be known until they become used more widely. As demonstrated with natalizumab, powerful inhibition of disease may go hand-in-hand with rare, but serious, infections or other side effects. The risks and benefits will have to be weighed by the patient and physician accordingly. Nevertheless, with the number of choices expanding, it is likely that most patients will eventually be able to find the right fit for their disease.
